# Silica‐coated magnetic nanobeads in a flow enrichment target capture Halbach (FETCH) magnetic separation system for circulating tumor cell enrichment

**DOI:** 10.1002/1873-3468.15094

**Published:** 2025-01-01

**Authors:** Peng Liu, Sitian He, Anouk Mentink, Pieter Hart, Yongjun Wu, Leon W. M. M. Terstappen, Pascal Jonkheijm, Michiel Stevens

**Affiliations:** ^1^ Department of Medical Cell Biophysics, TechMed Center, Faculty of Science and Technology University of Twente Enschede The Netherlands; ^2^ Laboratory of Biointerface Chemistry, Department of Molecules and Materials, TechMed Centre University of Twente Enschede The Netherlands; ^3^ College of Public Health Zhengzhou University China; ^4^ Department of General, Visceral and Pediatric Surgery Heinrich‐Heine University, University Hospital Düsseldorf Germany; ^5^ FETCH BV Deventer The Netherlands

**Keywords:** circulating tumor cells enrichment, FETCH system, magnetic nanobeads

## Abstract

Detecting circulating tumor cells (CTCs) is challenging due to their low presence and heterogeneity. Traditional methods using EpCAM‐based separation struggle with CTCs that have undergone epithelial‐mesenchymal transition, as this results in lower EpCAM expression. This study presents the use of silica‐coated magnetic nanobeads functionalized with streptavidin for CTC capture. Using the FETCH magnetic separation system, we validated the capture efficiency of our beads on tumor cells with varying EpCAM expression. Our beads showed superior capture rates for LNCaP (97%), PC3‐9 (91%), PC3 (23%), A549 (22%), and T24 (8%) cells compared to commercial MojoSort™ beads. Despite slightly higher nonspecific binding than CellSearch, our beads demonstrated improved sensitivity for EpCAMlow cells, suggesting they have promise for enhanced CTC capture.

## Graphical abstract

Silica‐coated magnetic nanobeads functionalized with streptavidin (NC@silica‐SA) efficiently capture circulating tumor cells (CTCs) with varying EpCAM expression. The FETCH system demonstrated superior sensitivity, especially for EpCAM^low^ cells, outperforming commercial beads and offering a promising tool for improved CTC detection.

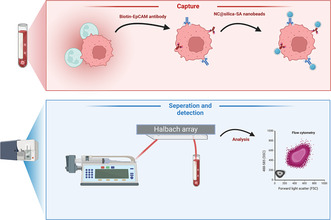

## Abbreviations

**ANOVA**, analysis of variance

**BMS**, biomagnetic solutions

**BSA**, bovine serum albumin

**CTC**, circulating tumor cell

**DMEM**, Dulbecco's modified Eagle medium

**EDC**, *N*‐(3‐dimethylaminopropyl)‐*N*′‐ethylcarbodiimide hydrochloride

**EG**, ethylene glycol

**EGFR**, epidermal growth factor receptor

**ELBS**, European Liquid Biopsy Society

**EpCAM**, epithelial cell adhesion molecule

**FACS**, fluorescence‐activated cell sorting

**FETCH**, flow enrichment target capture Halbach

**FITC**, fluorescein isothiocyanate

**FTIR‐ATR**, fourier‐transform infrared spectroscopy in attenuated total reflectance

**MES**, 2‐morpholinoethanesulfonic acid

**NaOAc**, sodium acetate

**NC**, nanoclusters

**PBS**, phosphate‐buffered saline

**PE**, phycoerythrin (used as a dye)

**S/TEM**, scanning transmission electron microscopy

**SA**, streptavidin

**SEM**, scanning electron microscopy

**tdEVs**, tumor‐derived extracellular vesicles

**TEM**, transmission electron microscopy

**VSM**, vibrating sample magnetometry

Circulating tumor cells (CTCs) are pivotal for diagnosing cancer, assessing therapeutic efficacy, and predicting prognosis [1]. However, their rarity in cancer patients' peripheral blood hinders detection [2]. Immunomagnetic enrichment is frequently used before immunofluorescence characterization [3, 4, 5]. Recently, we developed a flow enrichment target capture Halbach (FETCH) magnetic array to enhance the capture efficiency of CTC with low expression of the EpCAM antigen and compared commercially available streptavidin magnetic particles to determine the best performance for capture efficiency and low nonspecific binding using biotinylated anti‐EpCAM antibodies (P. Liu, S. He, P. Hart, A. van Kleef, S. Boxum, A. Mentink, Y. Wu, L.W.M.M. Terstappen, P. Jonkheijm & M. Stevens, unpublished data) [6]. To determine whether the capture efficiency could be further increased, preferably without an increase in nonspecific capture, we synthesized superparamagnetic beads and coated them with a layer of silicon dioxide to couple streptavidin to them and evaluated their CTC capture performance, as presented in this article.

## Materials and methods

### Cell culture

Prostate cancer cell lines PC3 (RRID: CVCL_0035) and LNCaP (RRID: CVCL_0395) along with bladder carcinoma T24 cell line (RRID: CVCL_0554) were sourced from ATCC (Manassas, VA, USA), while the prostate cancer (PC3‐9) cell line (RRID: CVCL_0035), a sub‐clone of the PC3 cell line, was generously provided by Immunicon (Huntingdon Valley, PA, USA). Lung adenocarcinoma (A549) cell line (RRID: CVCL_0023) was obtained from CANCER‐ID in the context of the European Liquid Biopsy Society (ELBS). LNCaP, PC3‐9, PC3, and A549 cells were cultured in RPMI 1640 medium (Gibco, Waltham, MA, USA). T24 cells were cultured in DMEM/Ham's F‐12 medium (Capricorn, Ebsdorfergrund, Germany). All culture media were supplemented with 10% fetal bovine serum (Sigma‐Aldrich, St. Louis, MO, USA), and 1% penicillin–streptomycin mixture (Lonza, Basel, Switzerland). The cells were maintained in a humidified atmosphere at 37 °C and were trypsinized using 0.05% trypsin–EDTA (Gibco, Waltham, MA, USA) upon reaching 70–80% confluence. Subsequently, cells were fixed with 1% formaldehyde in PBS and then stored at 4 °C. To quantify the expression of EpCAM antigens on the cell surface of these cell lines, our group previously used the PE conjugate VU1D9 (Sigma‐Aldrich) for cell staining. The exact experimental steps are described in detail in our previous article (P. Liu, S. He, P. Hart, A. van Kleef, S. Boxum, A. Mentink, Y. Wu, L.W.M.M. Terstappen, P. Jonkheijm & M. Stevens, unpublished data). The EpCAM expressions of LNCaP, PC3‐9, PC3, A549, and T24 cells were 628 400, 19 700, 7100, 6060, and 4877, respectively.

### Synthesis of carboxylic acid‐modified NC@silica (NC@silica‐COOH)

Fe_3_O_4_ nanoclusters (NC) were synthesized by dissolving 0.27 g of ferric chloride hexahydrate (FeCl_3_·6H_2_O), 0.05 g of sodium acrylate and 0.7 g of sodium acetate (NaOAc) in 10 mL of ethylene glycol (EG) under magnetic stirring. This mixture underwent a series of vacuum and nitrogen gas fill cycles, with vacuum intervals of 1 min. After stirring for 30 min, a homogeneous yellow solution was obtained and transferred to a Teflon‐lined stainless‐steel autoclave. This autoclave was purged with nitrogen gas for 5 min, sealed, and then heat‐treated at 200 °C for 10 h. After cooling to room temperature, the resulting Fe_3_O_4_ nanoclusters were magnetically washed six times alternately with ethanol and water and then resuspended in 9 mL of water. We obtained nanoclusters which consisted of different sized primary nanoparticles by changing the ratio of reactants.

For the synthesis of silica‐coated Fe_3_O_4_ nanoclusters (NC@silica), half of the obtained magnetic beads were dispersed in 33 mL of ethanol and stirred mechanically for 5 min. Following this, 1.5 mL of ammonia solution was added. After 30 min of additional stirring, 0.15 mL of tetraethyl orthosilicate (TEOS) diluted in 9 mL of ethanol was introduced. The reaction was allowed to proceed for 60 min. The resulting Fe_3_O_4_@SiO_2_ core/shell nanoclusters, or NC@silica, were magnetically separated and washed six times alternately with ethanol and water. During each washing phase, the particles were separated magnetically for 5 min, and after resuspension, they were sonicated for 10 s in a sonication bath. The beads were then resuspended in water.

The synthesized product was characterized using Fourier‐transform infrared spectroscopy in attenuated total reflectance mode (FTIR‐ATR, ALPHA II; Bruker, Billerica, MA, USA), vibrating sample magnetometry (VSM; Physical Property Measurement System (PPMS), Quantum Design, San Diego, CA, USA), and transmission electron microscopy (TEM, Spectra 300 S/TEM; Thermo Fisher Scientific, Waltham, MA, USA). The morphology and dimensions of the synthesized NCs and their NC@silica were characterized by Scanning Electron Microscopy (SEM) and Scanning/Transmission Electron Microscopy (S/TEM). To produce the carboxylic acid‐modified nanoclusters (NC@silica‐COOH), 100 mg of NC@silica was mixed in 10 mL of PBS (10 mm, pH 7.4) with 200 μL of carboxyethylsilanetriol. This mixture was stirred mechanically for 3 h and sequentially washed with water and ethanol as detailed in the previous steps.

### Synthesis of streptavidin‐conjugated nanobeads (NC@silica‐SA)

To synthesize NC@silica‐SA, we employed *N*‐(3‐dimethylaminopropyl)‐*N*′‐ethylcarbodiimide hydrochloride (EDC) (Merck, Darmstadt, Germany) and *N*‐hydroxysulfosuccinimide sodium salt (sulfo‐NHS) (Merck) to attach streptavidin to NC@silica‐COOH. For this, 5 mg of NC@silica‐COOH nanoparticles was taken and the supernatant was discarded after 5 min of magnetic separation. The beads were then washed twice with 6 mL of 50 mm 2‐morpholinoethanesulfonic acid monohydrate (MES) (pH 6.0) (Merck) containing 500 mm NaCl (Sigma‐Aldrich), mixed thoroughly to resuspend them in this new buffer. Following washing, the beads were magnetically separated, and the supernatant was discarded in preparation for the addition of the activation solution. Next, 3 mL of EDC at a concentration of 1.18 mg·mL^−1^ and 3 mL of 2.17 mg·mL^−1^ of sulfo‐NHS were prepared with 500 mm NaCl in 50 mm MES (pH 6.0). It is essential to note that before using the EDC and Sulfo‐NHS, they need to be equilibrated to room temperature to prevent low yield. Subsequently, the sulfo‐NHS and EDC solutions were added sequentially to the washed beads. After thoroughly mixing, the beads were incubated at room temperature for 15 min on the roller mixer. At the end of the reaction, the beads were placed on a magnet for 5 min to remove the supernatant and a solution of 0.55 mg·mL^−1^ streptavidin buffer (streptavidin powder dissolved in PBS solution) was added to the activated magnetic beads. The beads are then resuspended with a vortex stirrer and incubated on a vertical mixer for 2 h at room temperature. The beads were rinsed three times with 10 mm PBS (pH 7.4)/1% Tween 20. Five milliliter of 10 mm PBS/1% BSA was added, and the solutions were mixed for 2 h on the roller mixer to block the remaining reactive sites on the magnetic beads. The washing step is repeated three times and finally 500 μL of 10 mm PBS/1% BSA/0.02% sodium azide is added to resuspend the magnetic beads. The final concentration of the magnetic beads is 10 mg·mL^−1^.

### Magnetic response time

We initially established a calibration curve with NC@silica‐SA to evaluate their separation efficiency under various separation systems. We achieved this by diluting the beads in buffer and ensuring that their absorbance is below 1, which is the optimal range according to the Beer–Lambert law [7, 8]. By plotting the absorbance at 600 nm versus concentration, we established the relationship between concentration and absorbance. We then explored two different separation methodologies: first, with a BD IMag separator with varied separation durations, and secondly, with our flow‐through system at different flow rates. We measured the absorbance of supernatants after separation to determine the magnetic separation efficiencies of the beads.

### Optimization of the concentration of NC@silica‐SA

We optimized the concentration of magnetic beads by employing NC@silica‐SA prepared by using streptavidin from Merck. PC3‐9 cells were prestained with CellTracker orange (Thermo Fisher Scientific) and Hoechst 33342, and 12 mL of whole blood from healthy donors was stained with Hoechst 33342. Blood was collected from anonymized healthy volunteers in CellSave Preservative Tubes (Menarini Silicon Biosystems, Florence, Italy) at the University of Twente. In agreement with the Declaration of Helsinki, informed consent was obtained from all volunteers, and the blood collection procedure was approved by the local Medical Research Ethics Committee (METC Twente). All methods were carried out in accordance with relevant guidelines and regulations. Subsequently, approximately 52 500 PC3‐9 cells were pipetted into the 12 mL of Hoechst‐stained whole blood. Following this, 19.8 μL of 0.604 mg·mL^−1^ of the biotinylated anti‐EpCAM (clone VU1D9) antibody was added to the blood sample, and the mixture was incubated at 37 °C for 45 min. After the antibody incubation, the blood sample was divided into 1 mL aliquots and incubated with various concentrations of NC@silica‐SA (37.5, 75, 150, and 300 μg·mL^−1^) for 30 min, with three replicates for each concentration. The mixture was thoroughly mixed and then incubated at room temperature for 30 min on a roller mixer. Following incubation, the blood samples with added magnetic beads were separated by using the FETCH magnetic separation system. A 10 mL syringe (BD Biosciences, San Jose, CA, USA) was placed on a programmable syringe pump (Harvard Apparatus, Holliston, MA, USA) and connected to the outlet of a 0.6 mm Ibidi μ‐Slide channel using 1.0 mm inner diameter tubing (Ibidi GmbH, Gräfelfing, Germany), and the inlet of the channel was connected to the sample through another piece of tubing. When the pump was switched on, the sample was then pulled through the channel as the syringe was withdrawn. The channel was positioned directly under the Halbach magnet array, and the flow rate was set as 0.5 mL·min^−1^. After nearly complete aspiration of the samples, 1 mL of PBS/1% BSA buffer was added to rinse the chamber of uncaptured cells. This step was repeated once for another rinse. Once the suspension was fully aspirated, the pump was halted, and after removing the magnetic array, the captured fraction was flushed out using PBS/1% BSA. The captured portion was magnetically washed three times with PBS/1% BSA. Magnetically washed refers to a process where the magnetic bead‐cell complex is placed on a magnetic stand, and the supernatant is removed after 5 min. PBS/1% BSA is then added to resuspend the complex, followed by repeating the magnetic separation step. Finally, the magnetic bead‐cell complex is resuspended for flow cytometry measurement (FACS Aria II; BD Biosciences). This method ensures effective washing and isolation of the target cells.

The capture efficiency was calculated as:

$$\text{Capture efficiency}\;\left(\%\right)=\frac{\text{Captured cells}}{\text{Reference}}\times 100\%$$

where ‘Captured cells’ refers to either the number of white blood cells or tumor cells captured, and ‘Reference’ refers to either the number of white blood cells or tumor cells enumerated in the references, depending on whether the specific capture efficiency for tumor cells or the nonspecific capture efficiency for white blood cells is calculated.

The capture purity was calculated as:

$$\text{Capture purity}\;\left(\%\right)=\frac{\text{Captured tumor cells}}{\mathrm{All}\;\text{captured cells}}\times 100\%$$

All the samples were analyzed on a flow cytometer using a 50 μm filter to ensure the passage of single suspended cells. To set the gates for identification of captured tumor cells, we used unstained tumor cells as negative controls, while tumor cells stained with Hoechst 33342 and CellTracker orange served as positive controls. Moreover, the voltages of each channel were adjusted to be able to distinguish between stained and unstained cells. To exclude unstained cells and noncellular events, thresholds for the forward scattering channel and the 375–450/40 channel were established.

### Effect of different sources of streptavidin on capture efficiency

After determining the optimal concentration of NC@silica‐SA, we evaluated the capture efficiency of magnetic beads synthesized with streptavidin, which was purchased from Sigma (catalog number 85878; St. Louis, MO, USA) and Merck (catalog number SA101). The quality of streptavidin is evaluated based on its purity, the affinity and specificity of the combined biotin, its storage stability, the minimal nonspecific binding capacity, and other multifactorial factors. The quality level of 85 878 is 200 and that of SA101 is 100. The experimental procedure was the same as described in Optimization of the concentration of NC@silica‐SA section, and the concentration of magnetic beads used was 150 μg·mL^−1^.

### Characterization of NC@silica‐SA

We characterized the nanobeads using Zetasizer Nano ZS (Malvern Panalytical, Malvern, UK). To evaluate the stability of NC@silica‐SA (85878), we monitored the binding capacity of biotin‐FITC (Sigma‐Aldrich) after synthesis and different time points over 1 month. Initially, 10 μL of NC@silica‐SA beads at a concentration of 10 mg·mL^−1^ were taken, and the supernatant was removed through magnetic separation. The beads were then resuspended in 1 mL of 10 mm PBS containing 1% BSA. For the binding capacity tests, 100 μL of the prepared beads were mixed separately with various concentrations of biotin‐FITC, namely 0.443, 0.222, 0.111, 0.055, 0.028, 0.014, 0.007, 0.003, and 0.002 μg·mL^−1^. Each mixture was incubated on a rolling mixer for 30 min at room temperature to ensure proper interaction. After the magnetic separation step, 150 μL of the resulting supernatant was transferred to a 96‐well plate for fluorescence detection using an EnSpire Multimode Plate Reader (PerkinElmer, Shelton, CT, USA). A reference curve of different concentrations of biotin‐FITC was established, and the saturation concentration of biotin‐FITC was determined. The binding capacity (pmol·mg^−1^) is obtained by dividing the amount of biotin‐FITC at this saturation point by the amount of magnetic beads.

### Comparison of capture efficiency of FETCH and BD IMag™ cell separation magnet

To demonstrate that the FETCH magnetic separation system can further improve the capture efficiency of NC@silica‐SA on EpCAM^high^ cells and EpCAM^low^ cells, we used LNCaP (~ 628 400 EpCAM antigens) and T24 (~ 4877 EpCAM antigens) cells as validation cell lines to test the capture efficiency of the FETCH system and the BD IMag™ Cell Separation Magnet. The experimental procedure steps were the same as described in the Optimization of the concentration of NC@silica‐SA section, the difference was that after incubation with NC@silica‐SA, one session of the samples was separated using the Flow‐through Immunomagnetic CTC Enrichment system, and another session made with the same preparation process were separated using the Magnet Rack.

### Comparison with commercial beads

To evaluate the capture capacity of NC@silica‐SA for tumor cells, we implemented a comparative capture experiment. This experiment involved spiking blood samples with five cell lines, LNCaP (~ 628 400 EpCAM antigens), PC3‐9 (~ 19 700 EpCAM antigens), PC3 (~ 7100 EpCAM antigens), A549 (~ 6060 EpCAM antigens), and T24 (~ 4877 EpCAM antigens) to serve as representative models for high and low EpCAM expression cells. Five thousand cells from each cell line were spiked into 1 mL blood sample from healthy donors. The spiked samples were then incubated with the biotinylated‐VU1D9 antibody for 45 min at 37 °C. Subsequently, the samples were either incubated with Mojosort, BioMagnetic Solutions FerroSelect™ Ferrofluids (BMS) as described previously (P. Liu, S. He, P. Hart, A. van Kleef, S. Boxum, A. Mentink, Y. Wu, L.W.M.M. Terstappen, P. Jonkheijm & M. Stevens, unpublished data) or with our synthesized nanobeads at a final concentration of 150 μg·mL^−1^ and incubated on a rolling mixer. The remaining steps are the same as described in Optimization of the concentration of NC@silica‐SA section. The capture efficiency and capture purity are calculated as shown in Optimization of the concentration of NC@silica‐SA section.

### Capture efficiency of NC@silica‐SA with clinically relevant dilutions of cancer cells

To evaluate if the number of tumor cells in the samples affects the capture efficiency of NC@silica‐SA, we prepared aliquots of 7.5 mL blood samples from healthy donors and spiked them with 490, 490, 490, 57, 43, 53, 5, 6, and 5 CellTracker Red dye prestained LNCaP tumor cells. These cells were counted using flow cytometry and fluorescence microscopy. The spiked samples were then incubated with biotinylated‐VU1D9 antibody for 45 min at 37 °C. Following incubation, the samples were centrifuged at 800 RCF for 10 min without a break to remove the plasma and free antibodies. Subsequently, the samples were incubated on a rolling mixer with NC@silica‐SA at a final concentration of 150 μg·mL^−1^. The remaining separation steps were conducted as described in Optimization of the concentration of NC@silica‐SA section. After processing, the obtained cell suspensions were stained with Hoechst, and the numbers of LNCaP cells and leukocytes were enumerated by flow cytometry. The capture efficiency and capture purity were calculated as outlined in Optimization of the concentration of NC@silica‐SA section.

### Comparison with CellSearch system

Aliquots of 15 mL of blood were spiked with either ~ 500 LNCaP cells, ~ 500 PC3 cells, or ~ 500 T24 cells. All three cell lines were prestained with CellTracker red. Half of the blood (7.5 mL) was processed with the CellSearch system using the epithelial cell enrichment kit, and the other half was processed on the FETCH system using the NC@silica‐SA beads (as described in Optimization of the concentration of NC@silica‐SA section). Before incubating with NC@silica‐SA beads, the blood was incubated with biotinylated anti‐EpCAM antibody and washed once to remove unbound antibody. After processing the obtained cell suspensions, they were stained with Hoechst and the number of LNCaP cells, PC3 cells, T24 cells, and leukocytes were enumerated by flow cytometry.

### Statistical analysis

We used paired *t*‐tests to compare the differences between the BD IMag™ Cell Separation Magnet and the FETCH system, as well as the differences between the two brands of streptavidin. We used one‐way ANOVA to compare the capture efficiency of tumor cells between all eight magnetic beads. origin 2023 SR0 (OriginLab Corporation, Northampton, MA, USA) was used to conduct all statistical and correlation analyses using a 0.05 significance threshold.

## Results

### Workflow for synthesis and testing of magnetic nanoclusters for tumor cell enrichment

**Fig. 1:**
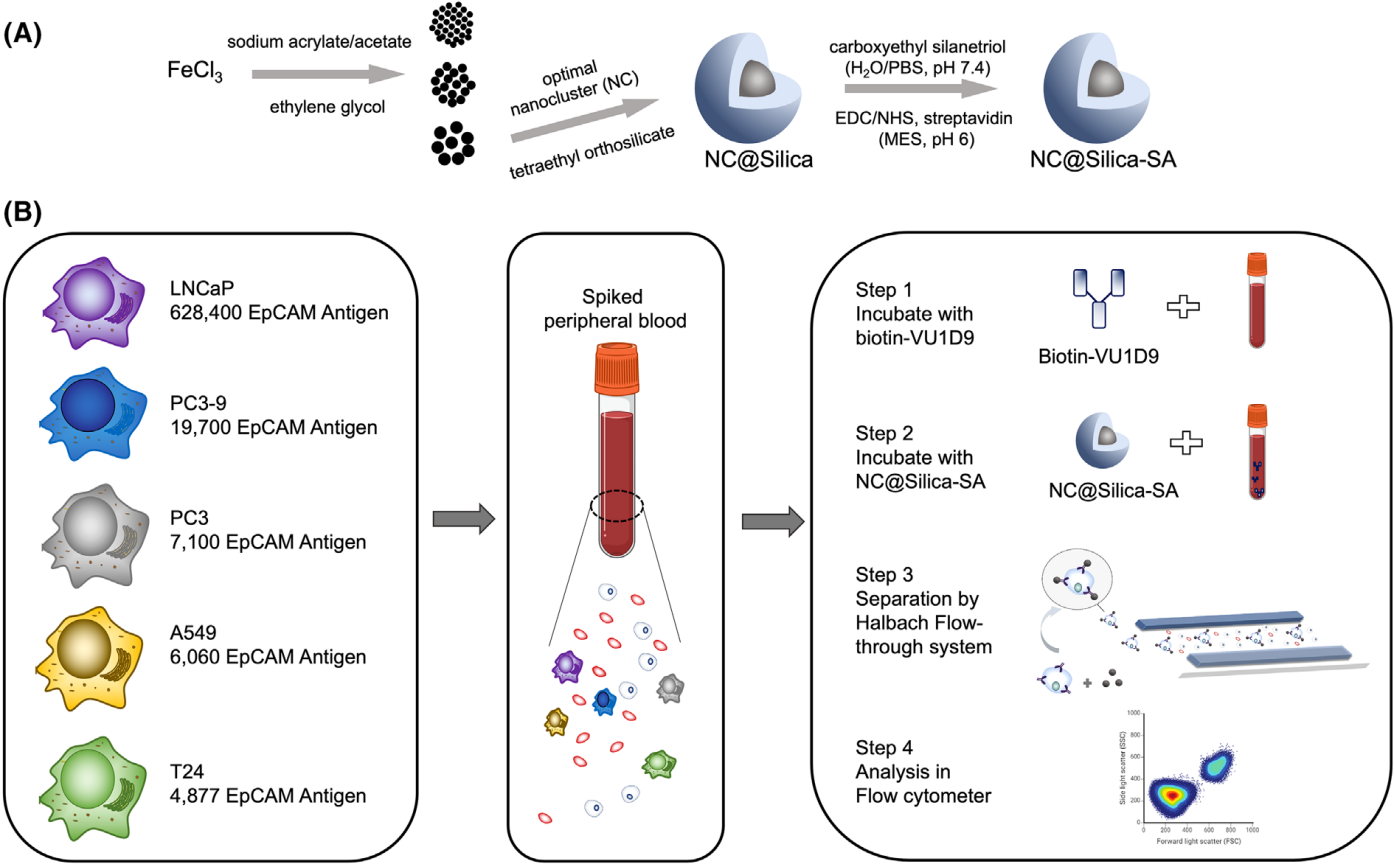
(A) Schematic representation of the synthesis of magnetic nanobeads modified with streptavidin. (B) Illustration of the process for enriching circulating tumor cells (CTCs) from spiked blood samples using the synthesized nanobeads. Tumor cells with varying EpCAM antigen expression levels are spiked into blood samples. Each 1 mL blood sample is incubated with biotinylated anti‐EpCAM antibodies (Step 1), followed by incubation with streptavidin‐coated magnetic nanobeads (NC@Silica‐SA) (Step 2). The samples are then processed through the Flow‐through Immunomagnetic CTC Enrichment system (Step 3). Finally, the enriched cells in the suspension are enumerated using flow cytometry (Step 4). Created with BioRender.com.

Figure 1 provides a graphical depiction of the synthesis and performance evaluation of the beads within the FETCH system. This evaluation involved the utilization of blood samples spiked with tumor cells exhibiting varying EpCAM antigen profiles, thereby enabling a thorough assessment of the beads' efficacy in differentiating and capturing these distinct cellular entities.

### Particle characterization

**Fig. 2:**
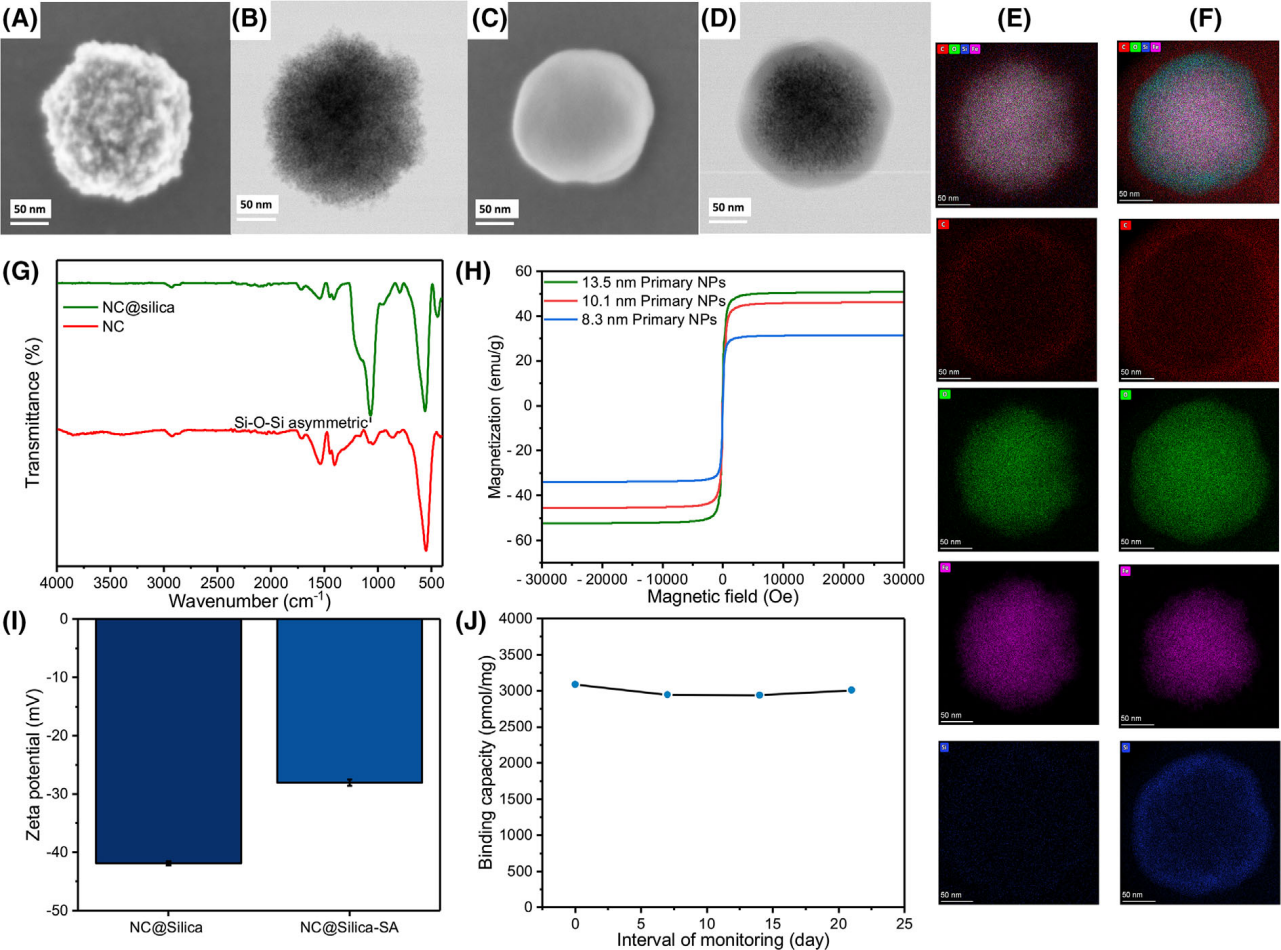
(A) Scanning electron microscopy (SEM) image of Fe_3_O_4_ nanoclusters (NC). (B) Scanning transmission electron microscopy (STEM) image of NC. (C) SEM image of silica‐coated Fe_3_O_4_ nanoclusters (NC@silica). (D) STEM image of NC@silica. (E) TEM‐EDS elemental mapping of NC showing the distribution of carbon, oxygen, silicon, and iron. (F) TEM‐EDS elemental mapping of NC@silica showing the distribution of carbon, oxygen, silicon, and iron with an increased area of oxygen and silicon distribution due to the silica coating. (G) Fourier‐transform infrared spectroscopy in attenuated total reflectance mode (FTIR‐ATR) spectra of NC and NC@silica showing a pronounced Si–O–Si asymmetric peak at 1070 cm^−1^, indicating successful silica coating. (H) Vibrating sample magnetometry (VSM) curve of primary nanoparticles before and after coating with the silica layer, demonstrating the superparamagnetic nature of NC@silica. (I) Zeta potential measurements of NC@silica and NC@silica‐SA showing the shift from −41.85 to −28.01 mV after streptavidin modification, confirming successful conjugation. Columns represent the mean Zeta potential, whiskers indicate mean ± SD, *n* = 5. (J) Stability of NC@silica‐SA over 20 days, demonstrating excellent biotin binding stability.

The morphology and dimensions of the magnetic nanobeads were characterized using electron microscopy (Fig. 2A–D). The NCs exhibited an average size of 172 nm and had a rough surface, as seen in Fig. 2A. This can be attributed to the fact that these clusters were aggregates of nanoparticles. S/TEM images revealed the nanoclusters as solid spheres, as shown in Fig. 1B. The thickness of this silica shell was found to be approximately 9 nm, as observed using TEM (Fig. S1). Figure 2E,F showed that iron and oxygen were distributed throughout the entire sphere. NC@silica has a silica ring and the area of the oxygen distribution increased compared to the NC without silica. In the ATR‐FTIR spectrum, a pronounced Si–O–Si asymmetric peak was observed at 1070 cm^−1^ (Fig. 2G). This peak serves as additional proof of the successful coating of the silica shell. To determine the magnetism property, we conducted VSM. The intersection of the VSM curve with the zero point in Fig. 2H substantiates the superparamagnetic nature of the NC@silica. Furthermore, the saturation magnetization increased along with an increase in the size of the primary nanoparticles, and the highest saturation magnetization was obtained with the nanoclusters consisting of 13.5 nm primary NPs, which was qualified to be 52.4 emu·g^−1^. This high saturation magnetization indicated the potential utility of these nanoclusters in applications demanding high magnetic responsiveness.

The biotin–streptavidin system is a commonly used coupling method used for capturing biotin antibody‐labeled tumor cells [9, 10, 11]. By coupling streptavidin to the surface of carboxylic acid‐modified magnetic beads, this system can capture biotinylated antibody‐labeled tumor cells. The EDC/sulfo‐NHS reaction facilitates the coupling of the amino group of streptavidin to the carboxyl groups on the beads. As depicted in Fig. 2I, the zeta potential of NC@Silica‐COOH shifted from −41.85 to −28.01 mV following streptavidin modification, confirming the successful conjugation of streptavidin onto the surface of NC@Silica‐COOH. The stability of NC@silica‐SA plays a crucial role in ensuring the accuracy of experimental results, the duration of the experiments, and their overall cost‐effectiveness. Therefore, it is essential to prioritize and ensure the stability of these magnetic beads. Figure 2J demonstrates that the magnetic beads exhibited excellent biotin binding over 20 days.

### Optimization of the concentration of NC@silica‐SA and selection of streptavidin

To establish an effective capture method for CTCs using NC@silica‐SA, we conducted a series of experiments to optimize the relevant parameters (*n* = 3). To enhance the optimization efficiency, we specifically selected PC3‐9 cells with medium‐low EpCAM expression as the target for detection.

**Fig. 3:**
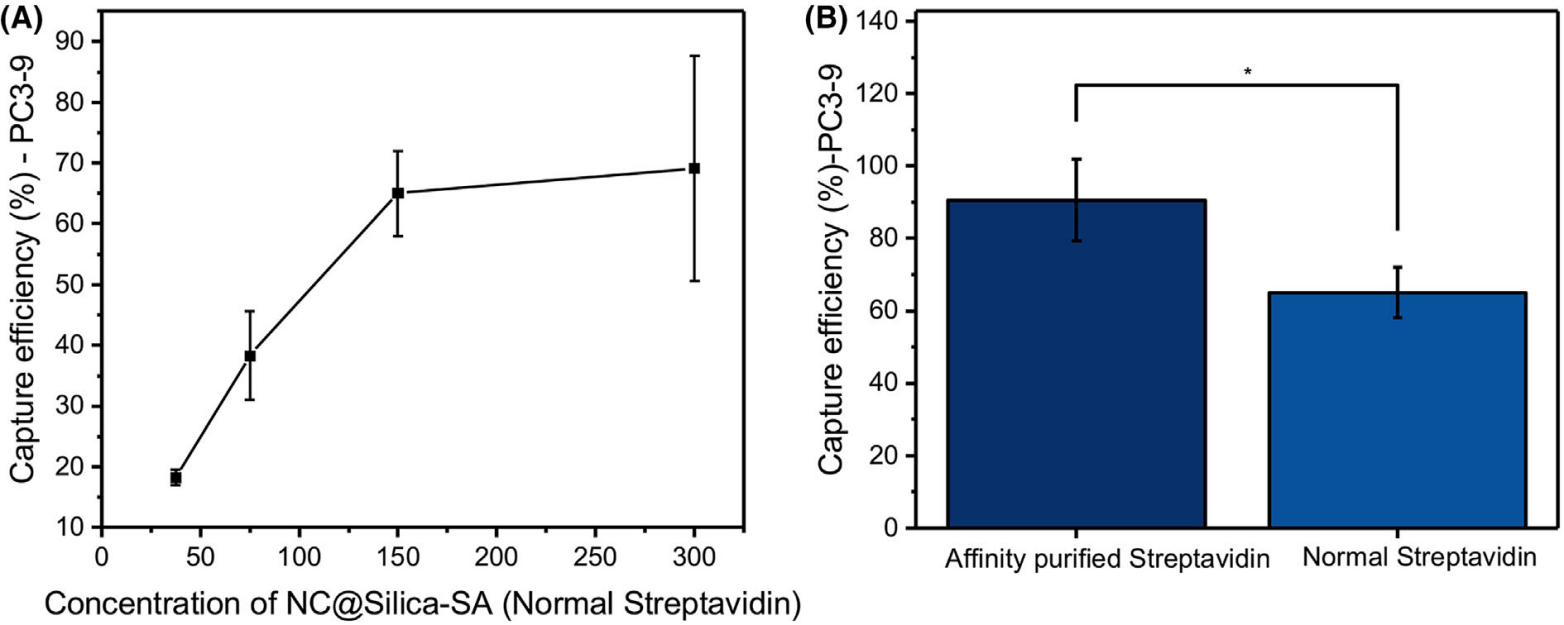
(A) Capture efficiency of NC@silica‐SA for PC3‐9 cells at different concentrations of NC@silica‐SA (using normal streptavidin), *n* = 3. The capture efficiency increases with the concentration of NC@silica‐SA beads, reaching a plateau at 150 μg·mL^−1^. (B) Comparison of capture efficiency for PC3‐9 cells between NC@silica‐SA 85878 (affinity‐purified streptavidin) and NC@silica‐SA 101 (normal streptavidin), *n* = 3. Columns represent the mean capture efficiency, whiskers indicate mean ± SD, and horizontal lines within the whiskers represent the median. The paired *t*‐test was used to compare the mean differences in capture efficiency (%) of PC3‐9 between affinity‐purified streptavidin and normal streptavidin. *P < 0.05.

From the findings presented in Fig. 3A, we observed that the capture efficiency of NC@silica‐SA for PC3‐9 cells increased as the used concentration of beads increased. However, it was evident that the capture efficiency eventually reached a saturation point at a concentration of 150 μg·mL^−1^. Therefore, based on these results, we utilized the magnetic beads at a concentration of 150 μg·mL^−1^ for all subsequent experiments.

The quality level of streptavidin plays a crucial role in its ability to effectively capture biotin antibody‐labeled cells. To study the effect on the beads' capturing ability, we compared streptavidin from two different commercial sources. Figure 3B also revealed that using the affinity‐purified streptavidin (85878) to synthesize NC@silica‐SA resulted in a higher capture efficiency for PC3‐9 cells (*t*‐test, *P* < 0.05) than normal streptavidin (SA 101). Therefore, affinity‐purified streptavidin (85878) has been chosen for the subsequent experiments.

### Magnetic response time of NC@silica‐SA

The beads' magnetization highly affects the separation duration, and we can investigate the beads' magnetic response time on different separation configurations to estimate the needed separation duration to entirely separate the beads.

**Fig. 4:**
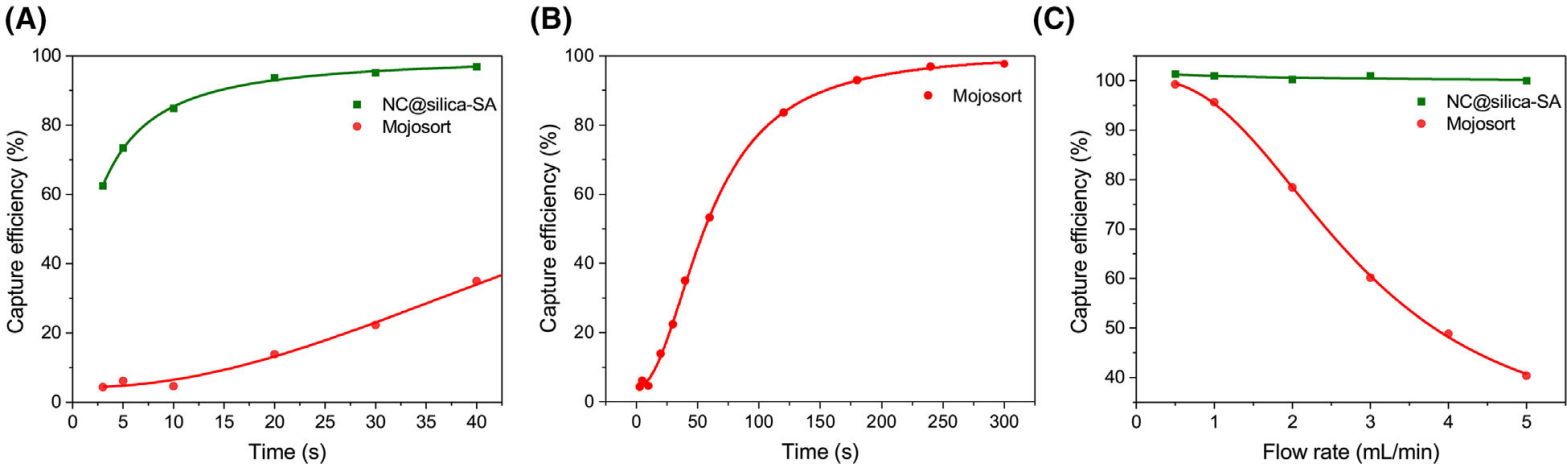
(A) Capture efficiency of NC@silica‐SA beads and Mojosort beads on the BD IMag magnet separator over a duration of 40 s. The NC@silica‐SA beads show rapid capture, reaching 63% efficiency in 3 s and 97% in 40 s, while the Mojosort beads reach only 35% in the same period. (B) Extended capture efficiency of Mojosort beads on the BD IMag magnet separator over 5 min, ultimately achieving 98% efficiency. (C) Capture efficiency of NC@silica‐SA beads and Mojosort beads on the Flow‐through Immunomagnetic CTC Enrichment system at various flow rates. NC@silica‐SA beads maintain high efficiency at flow rates below 5 mL·min^−1^, while MojoSort beads show decreased efficiency as the flow rate increases, dropping to 40% at 5 mL·min^−1^.

We first tested it on BD IMag magnet separator, since this has been widely used by researchers. Figure 4A demonstrates how efficiently the beads can be captured by the BD IMag magnet separator over time. 63% of the NC@silica‐SA were captured by the configuration in 3 s, while, in contrast, only 5% of the Mojosort beads were captured. The capture efficiency of NC@silica‐SA saturated gradually, reaching 97% in 40 s. At this point, the suspension became transparent. At the same time point, only 35% of the Mojosort beads were captured. To obtain a full picture of the capture situation of Mojosort over time, we monitored it for 5 min, at which point 98% capture was observed (Fig. 4B). The pictures taken during the separation can be found in Fig. S2A,B.

As Fig. 4C shows, we also tested the influence of the flow rate on the capture efficiency of the FETCH separation system. As can also be seen in the photos of the channels after separation in Fig. S2C, when using the NC@silica nanobeads, no beads reached the channels exit at speeds below 5 mL·min^−1^. On the other hand, starting from 0.5 mL·min^−1^, with the increase of the flow rate, part of the Mojosort beads cannot be kept in the channel anymore, and we see clearly from the picture (Fig. S2D) that the beads traveled through the channel from inlet to outlet. The capture efficiency decreased from 99% at 0.5 mL·min^−1^ to 40% at 5 mL·min^−1^. The physical diagram of our magnetic beads and Mojosort beads optimized for separation on a magnetic stand and FETCH system is shown in Fig. S2C,D.

### Comparison of FETCH and BD IMag™ cell separation magnet

**Fig. 5:**
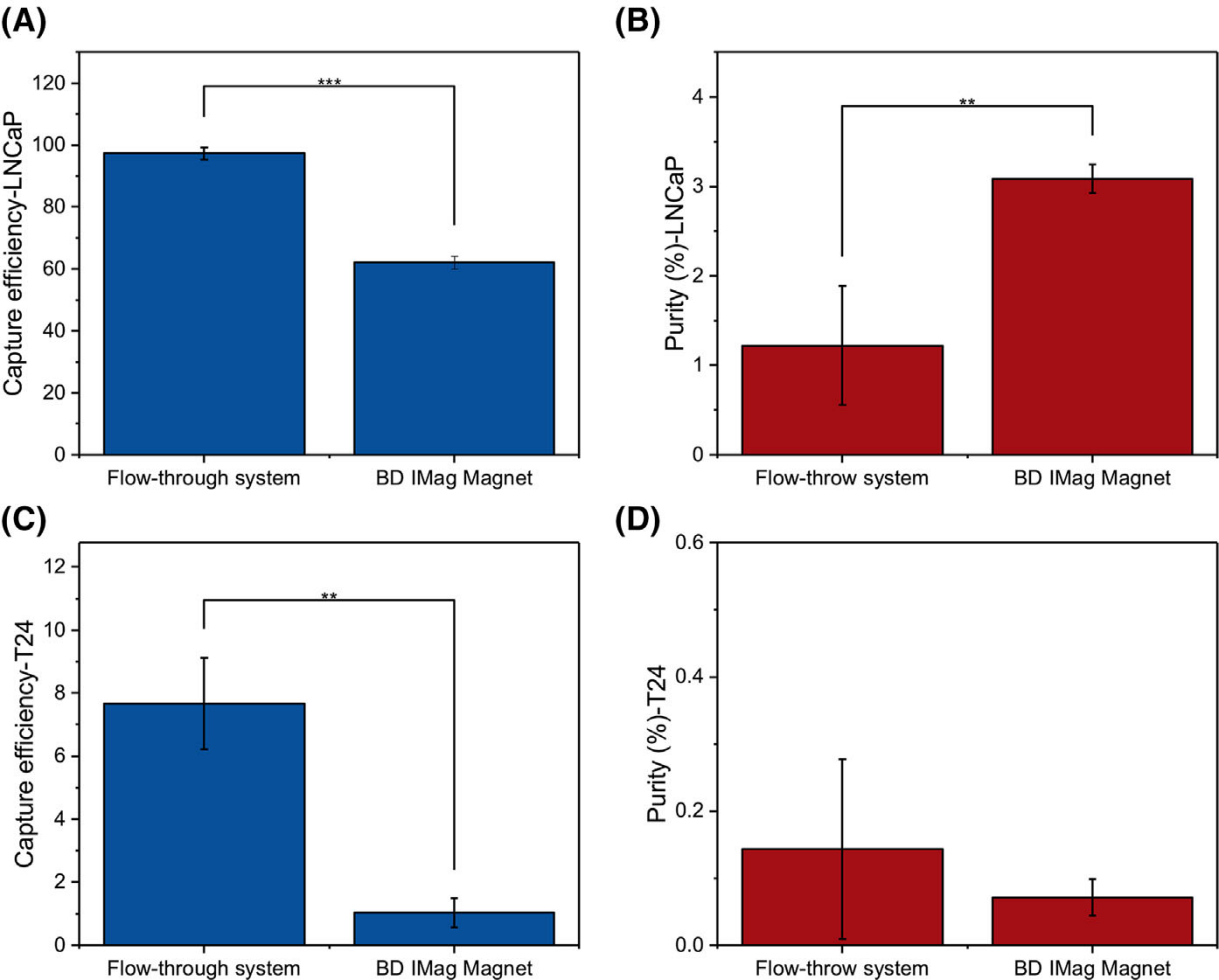
Comparison of the flow‐through immunomagnetic CTC enrichment system and magnet rack for LNCaP cells (A, B) and T24 cells (C, D), showing capture efficiency and capture purity, *n* = 3. Columns indicate the mean, whiskers represent mean ± SD, and horizontal lines within the whiskers indicate the median. All experiments employed the paired *t*‐test to determine whether there were statistically significant differences between the two groups. **P < 0.01, ***P < 0.001.

The FETCH magnetic separation system has the potential advantage of being applied to large‐volume samples because it separates magnetic samples in a continuous flow mode. Apart from that, it also has the advantage of an enhanced magnetic field. We selected two of the five cell lines with the highest and lowest EpCAM expression levels, LNCaP and T24 cell lines to compare the differences when using the FETCH system and magnet rack (*n* = 3). We found that, as shown in Fig. 5A,C, the FETCH flow system can significantly improve the capture efficiency of LNCaP and T24 cells compared with BD IMag™ Cell Separation Magnet. Specifically, the capture efficiency of LNCaP increased from 62% to 97% (*P* < 0.001), while that of T24 increased from 1.0% to 7.7% (*P* < 0.01). However, for EpCAM^high^ LNCaP cells, the capture purity was reduced from 3.1% to 1.2% (*P* < 0.01) when moving from BD IMag™ Cell Separation Magnet to the FETCH flow system (Fig. 5B). No significant change in the purity of T24 cells was found between the BD IMag™ Cell Separation Magnet (capture purity is 0.07%) and the FETCH system (capture purity is 0.14%) (Fig. 5D). Since thousands of CTCs are seldomly encountered in clinical samples, we therefore reduced the number of spiked LNCaP cells by tenfold to assess whether capture efficiency would be affected by a lower tumor cell concentration. The results demonstrated that the capture efficiency remained unchanged, as illustrated in Fig. S3.

### Comparison between NC@silica‐SA and commercially available magnetic beads

In our previous work, we evaluated the performance of eight commercially available magnetic beads and identified three that performed best (P. Liu, S. He, P. Hart, A. van Kleef, S. Boxum, A. Mentink, Y. Wu, L.W.M.M. Terstappen, P. Jonkheijm & M. Stevens, unpublished data). We utilized two top performers for a comparative analysis with our NC@silica‐SA beads. The gating for flow cytometry was performed identical to our previously reported methods.

**Fig. 6:**
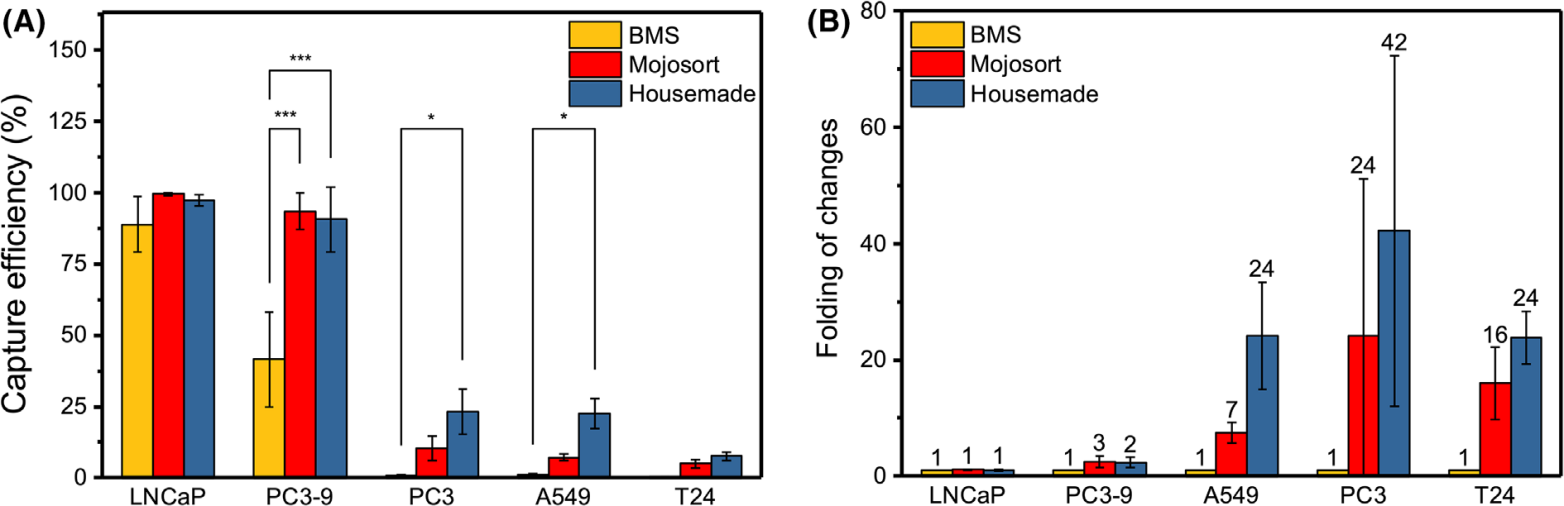
Aliquots of 1 mL of EpCAM‐biotin added tumor cells were spiked into blood and incubated with three types of beads, followed by separation using the flow‐through device. (A) Capture efficiency of five cell lines (LNCaP, PC3‐9, PC3, A549, and T24) with different EpCAM expression levels, *n* = 3. (B) Fold change in capture efficiency compared to the BMS ferrofluids, *n* = 3. Columns represent the mean, whiskers indicate mean ± SD, and horizontal lines within the whiskers represent the median. All experiments employed the paired *t*‐test to determine whether there were statistically significant differences between the two groups. *P < 0.05, ***P < 0.001.

Figure 6A presents the capture efficiencies for LNCaP, PC3‐9, A549, PC3, and T24 cell lines across different bead types. For EpCAM^high^ LNCaP cells, all beads demonstrate over 89% capture efficiency and there is no significant difference between them. Meanwhile, for EpCAM^Medium^ PC3‐9, the efficiency ranges from 42% to 93%, and there is no significant difference between NC@silica‐SA beads and Mojosort, however, both of them significantly perform better than BMS ferrofluids (*t*‐test, *P* < 0.001).

These findings align with our previous results, acknowledging minor variability. As for the EpCAM^low^ cell lines, the advantages of the NC@silica‐SA beads start to show. For all three cell types, Mojosort beads did not show a significant difference in capture efficiency compared to BMS ferrofluids. However, the NC@silica‐SA beads demonstrated a statistically significant increase in capture efficiency on A549 and PC3 cells compared to BMS ferrofluids (*t*‐test, *P* < 0.05). This result can be attributed to the high magnetization of the NC@silica‐SA beads, facilitating their binding to target cells and enabling their attraction to an external magnet, even when relatively few beads are bound to the cell surface. Furthermore, the purification method of SA could affect the capture efficiency as well. It is however not known which kind of streptavidin is used in the production of commercial beads. To demonstrate the improvement directly, we created Fig. 6B, which is the capture efficiency of Mojosort and NC@silica‐SA beads divided by the capture efficiency of BMS beads. Upon analyzing Fig. 6B, it becomes apparent that the capture efficiency for LNCaP cells (628 400 EpCAM) with our beads remains comparable to that of the other two bead types. Surprisingly, as the EpCAM content decreases, our beads distinctly showcase their advantages. Specifically, on PC3‐9 cells (19 700 EpCAM), our beads exhibited twice the capture efficiency compared to the other bead types. Remarkably, on A549 cells (6060 EpCAM antigens), our beads demonstrated a 24‐fold increase in capture efficiency. Moreover, on PC3 cells (7100 EpCAM), our beads displayed a remarkable 42‐fold improvement in capture efficiency. Finally, for T24 cells (4877 EpCAM), our beads exhibited a 24‐fold increase in capture efficiency compared to the other bead types.

### Capture efficiency of NC@silica‐SA with clinically relevant dilutions of cancer cells

**Fig. 7:**
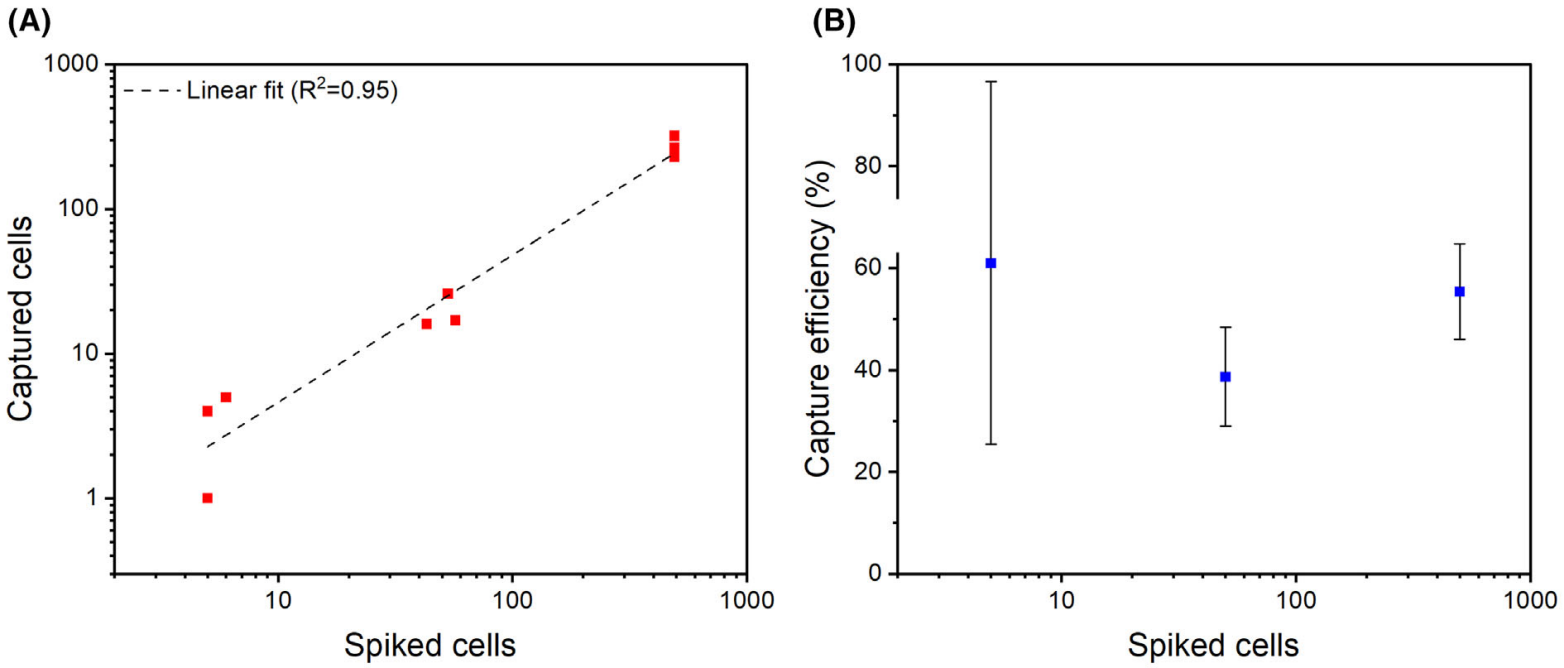
Relationship between spiked cells and captured cells, and capture efficiency of NC@silica‐SA nanobeads at different concentrations of spiked LNCaP tumor cells in 7.5 mL blood samples. (A) Scatter plot of captured cells versus spiked cells with a log–log scale. A linear fit of the log‐transformed data (dashed line) shows a strong correlation with an *R* ^2^ value of 0.95. (B) Capture efficiency (%) versus spiked cells on a log scale. Error bars represent the standard deviation (*n* = 3).

A precise number of LNCaP tumor cells 490, 490, 490, 57, 43, 53, 5, 6, and 5 were spiked into 7.5 mL blood samples from healthy donors to evaluate the capture efficiency of NC@silica‐SA nanobeads at different tumor cell levels. A control with no spiked tumor cells was also performed. The results are presented in Fig. 7. In three separate experiments, the capture efficiency of the approximately 500 LNCaP cell spike was 54.08%, 46.73%, and 65.31% (mean 55.37, SD = 9.35), the capture efficiency of the approximately 50 LNCaP cell spike was 29.82%, 37.21%, and 49.06% (mean 38.7, SD = 9.7), and the capture efficiency of the approximately 5 LNCaP cell spike was 80.00%, 83.33%, and 20.00% (mean 61.11, SD = 35.64). In the samples with no LNCaP cells spiked, no cells were detected in the LNCaP cell gate.

### Comparison between NC@silica‐SA and CellSearch

**Fig. 8:**
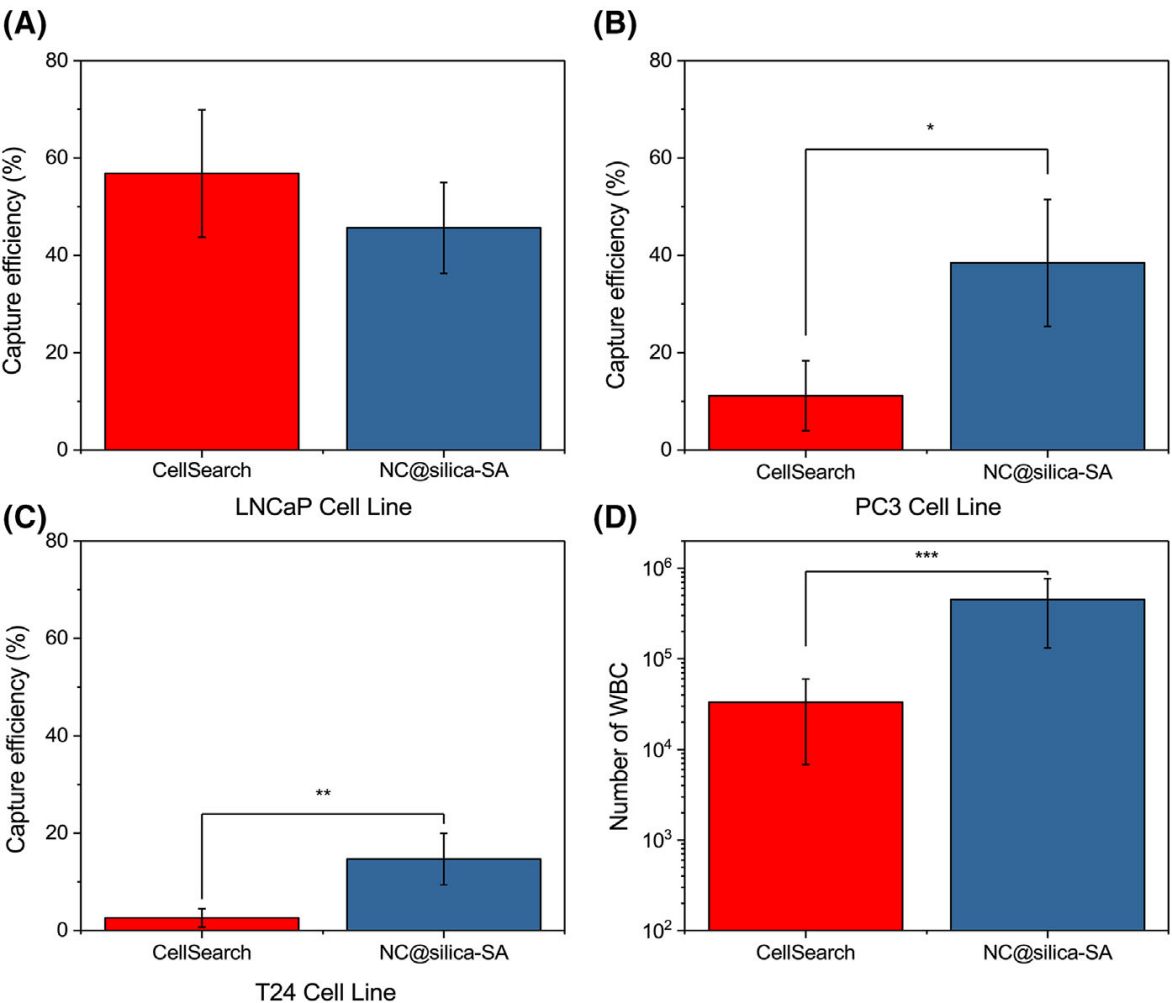
LNCaP cells, PC3, and T24 cell lines were spiked into 15 mL of blood and then divided into two parts to compare the capture efficiency of our method and the CellSearch method. Each method used 7.5 mL of blood. (A) Capture efficiency of LNCaP cells. (B) Capture efficiency of PC3 cells. (C) Capture efficiency of T24 cells. (D) Comparison of the number of white blood cells captured by our method and the CellSearch method in 7.5 mL of blood. Columns indicate the mean, whiskers represent mean ± SD, and horizontal lines within the whiskers indicate the median. *N* = 5. All experiments employed the paired *t*‐test to determine whether there were statistically significant differences between the two groups. *P < 0.05, **P < 0.01, ***P < 0.001.

From the aforementioned results, it was observed that our streptavidin‐coated magnetic beads, coupled with the FETCH system, exhibited a higher capture efficiency for cells with low EpCAM antigen density. We evaluated this in a comparative analysis with the CellSearch system for enriching LNCaP (628 400 EpCAM), PC3 (7100 EpCAM), and T24 (4877 EpCAM) cells introduced into 7.5 mL of blood. As illustrated in Fig. 8, our method showed greater capture efficiency for cells expressing lower antigen levels compared to the CellSearch system. For LNCaP cells, our magnetic beads and CellSearch magnetic beads showed capture efficiencies of 46% and 57%, respectively, without any statistically significant difference. However, when it comes to PC3 cells, our capture efficiency surpassed that of CellSearch significantly; ours were at 38% compared to CellSearch's 11%, demonstrating a marked superiority (*P* < 0.05). Similarly, for T24 cells, our capture efficiency measured 15%, while CellSearch achieved only 3%, indicating a notably higher capture efficiency of our magnetic beads over CellSearch beads (*P* < 0.001). Nevertheless, the increased sensitivity of our beads may come with a trade‐off in higher nonspecific adsorption. Our beads exhibited nonspecific adsorption of around 10^5^ leukocytes, whereas CellSearch showed about 10^4^. Additionally, the number of nontarget cells captured by our beads was tenfold higher than theirs, highlighting an area that requires further optimization.

## Discussion

The detection of the presence of tumor cells in blood using immunomagnetic enrichment targeting EpCAM introduced in 1998 initiated the development of the CellSearch system [12, 13]. The initial clinical studies performed with this system showed a strong correlation between clinical outcomes and the presence of CTC [14, 15] and led to numerous clinical research studies and the development of novel technologies to detect and characterize CTC [16, 17]. The CellSearch system demonstrates high efficiency in detecting tumor cells with high expression of EpCAM. However, due to the heterogeneity among tumor cells, some cells inherently express low levels or lack expression of EpCAM [18]. Alternatively, it is possible that tumor cells which originally exhibited high EpCAM expression undergo epithelial–mesenchymal transition, resulting in decreased EpCAM expression levels, thereby preventing detection by the CellSearch system [19]. In the CellSearch system, this is partly overcome by so‐called “controlled aggregation”, which increases the capture of tumor cells with low EpCAM antigen density [13, 19]. Another approach is to increase the attraction of cells with a low number of magnetic particles this is, for example, obtained with the FETCH magnetic enrichment system [20]. This study explores the enhanced separation of cells with low EpCAM antigen density through increased magnetic properties of the used beads. For this, we first optimized the size of the particles and covered them with a silica shell to obtain particles with an average size of 172 nm and a silica shell of approximately 9 nm. The size of the Fe_3_O_4_ nanoclusters within the 172 nm particle was optimized to obtain the strongest magnetic properties. We choose to couple streptavidin to the beads as it will allow us to explore the addition of targets beyond EpCAM. The final NC@silica‐SA beads are around 200 nm. To reduce nonspecific binding to leukocytes, we used BSA as a blocking agent during the separation process. While this reduced unwanted interactions, some nonspecific leukocyte binding persists. Further optimization of the bead surface and buffer conditions is needed to improve CTC capture specificity. Consistent binding stability of beads is essential for applications. We monitored the binding stability over 20 days and observed that the capture efficiency of the beads remained stable in our experiments conducted for over 6 months. Further testing across longer durations could provide additional insights into their suitability for long‐term use. We first compared it with three commercially available magnetic beads of similar size in the FETCH enrichment system to capture cells from cell lines with different EpCAM densities in 1 mL blood volumes. CTCs are rare in clinical samples. However, in this manuscript, the use of a higher number of spiked cells was driven by the aim to ensure statistical significance and reduce variability in our experimental outcomes, which can be more pronounced at extremely low cell counts. We used five cell lines with different EpCAM expressions to mimic the heterogeneity of clinical samples and prevent bias from only using high EpCAM expression cell lines. The peripheral blood has been chosen for spike‐in, instead of buffer solutions, to reflect the effect of other blood cells, proteins, and viscosity from blood. Subsequently, three tumor cell lines with varying EpCAM densities were spiked into 7.5 mL blood, and the performance of our method was compared with the clinically validated CellSearch system. The results indicated that for cells expressing low antigen levels, our method exhibited higher capture efficiency compared to the CellSearch system. However, for cell lines like T24, which have low EpCAM expression, our capture efficiency only reached 38%, indicating that there is still need improvement. Many researchers are dedicated to finding alternative markers to replace EpCAM. For instance, Bendre *et al*. [21] utilized folic acid as a marker to capture CTCs. Jiang *et al*. [22] employed gene editing technology to express a tri‐target molecule consisting of anti‐EpCAM, EGFR, and Her2 scFv on Jurkat membranes to enhance CTC capture efficiency. Additionally, we also attempted to incorporate a new recognition biomarker, rVAR2, alongside EpCAM antibodies. Our results demonstrated that combining rVAR2 with EpCAM antibodies further improved the capture efficiency for cells with low EpCAM expression [23]. Apart from the capture efficiency, our method also showed an increase in the background signal from leukocytes, which requires further optimization. This could potentially be achieved by optimizing the buffer solutions utilized during incubation and separation steps or modifying beads with some new materials. Some researchers have attempted to coat the magnetic beads with cells homologous to leukocytes in order to reduce nonspecific adsorption of leukocytes [22, 24]. However, using living cells as a material for constructing such systems has certain limitations, as the complexity of cell membrane components makes it challenging to precisely control batch‐to‐batch variability [25]. In order to address this issue, researchers have recently begun exploring the use of well‐defined, commercially available materials (such as human serum albumin) to create controlled‐structure membrane substitutes, aiming to achieve the same effect of reducing nonspecific adsorption [26]. We also conducted experiments where approximately 500, 50, 5, and 0 LNCaP cells were spiked into 7.5 mL of blood, resulting in consistent capture efficiencies compared to later comparison experiments with the CellSearch system. Although this confirmed the consistency and robustness of our protocol, large standard deviations were observed at low target cell concentrations, as expected. Additionally, nonparametric tests, such as the Mann–Whitney *U* or Kruskal–Wallis, could be considered in future studies to further explore variability, particularly at low cell numbers, where assumptions of normality may not hold. Even though we have attempted to use realistic spiked‐in samples, these can never completely represent clinical samples. It is therefore important to validate this method using clinical samples. Here, we evaluated the ability of the NC@silica‐SA beads to capture cells with a large range in EpCAM antigen density. Whether or not the NC@silica‐SA beads also can improve the enrichment of tumor‐derived extracellular vesicles (tdEVs) in either plasma or cell fraction of blood remains to be explored. The presence of streptavidin on the NC@silica‐SA beads allows us to evaluate different compositions of biotinylated probes that recognize cancer cells but are not present in hematopoietic cells. Exploring the addition of for example biotinylated Spycatcher‐recombinant VAR2CSA malaria protein [27, 28] to biotinylated EpCAM blood samples of cancer patients in which a relatively low number of CTC are detected targeting EpCAM such as nonsmall cell lung cancer, ovarium, pancreas, and colorectal cancer [29, 30] will show whether or not broadening of the target antigens and improving the capture efficiency of low target antigens will improve the enrichment of tumor cells in the blood.

## Conclusion

The FETCH system is a user‐friendly device equipped with a Halbach array, which enhances CTC capture efficiency through stronger magnetism compared to conventional magnetic racks. We synthesized magnetic nanoparticles (NC@silica‐SA beads) with large clusters for maximal magnetic force, coated them with silicon, and coupled them with streptavidin. These beads were tested using five low‐EpCAM expressing cell lines (LNCaP, PC3‐9, A549, PC3, and T24) spiked into healthy blood and enriched through the FETCH system. The beads significantly improved capture efficiency of low EpCAM tumor cells compared to the CellSearch system, though with increased leukocyte co‐enrichment. Further optimization of buffer conditions may improve CTC purity, and clinical testing will reveal whether the enhanced magnetic properties of the beads lead to a higher CTC detection rate.

## Conflicts of interest

MS is a co‐owner of FETCH BV. The other authors declare no potential conflict of interest.

## Author contributions

PL and SH contributed to writing‐original draft preparation; PL, PH, AM, and YW contributed to methodology; MS, LWMMT, and PJ contributed to writing—review and editing. All authors have read and agreed to the published version of the manuscript.

### Peer review

The peer review history for this article is available at https://www.webofscience.com/api/gateway/wos/peer‐review/10.1002/1873‐3468.15094.

## Supporting information


**Fig. S1.** Transmission electron microscopy (TEM) images of NC@silica beads.
**Fig. S2.** (A) Physical images depict NC@silica‐SA beads being magnetically captured at various time intervals. (B) A physical image illustrates Mojosort beads being magnetically captured at different time points. (C) Physical images show NC@silica‐SA beads captured by the FETCH system at different time intervals. (D) Physical images display Mojosort beads captured by the FETCH system at various time points.
**Fig. S3.** Capture efficiency comparison of spike in 500 and 5000 LNCaP cells.

## Data Availability

The data supporting in manuscript are available in the article and Supporting Information. All other data are available from the corresponding author upon reasonable request.
