# Supplementary material for: Silica‐coated magnetic nanobeads in a flow enrichment target capture Halbach (FETCH) magnetic separation system for circulating tumor cell enrichment
Source: FEBS Lett. 2025 Jan 1;599(5):724–38. doi: 10.1002/1873-3468.15094 (PMC11891416; doi:10.1002/1873-3468.15094)
Supplement: Supplementary file 1 — Fig. S1. Transmission electron microscopy (TEM) images of NC@silica beads. Fig. S2. (A) Physical images depict NC@silica‐SA beads being magnetically captured at various time intervals. (B) A physical image illustrates Mojosort beads being magnetically captured at different time points. (C) Physical images show NC@silica‐SA beads captured by the FETCH system at different time intervals. (D) Physical images display Mojosort beads captured by the FETCH system at various time points. Fig. S3. Capture efficiency comparison of spike in 500 and 5000 LNCaP cells. [file FEB2-599-724-s001.docx]

**Silica-coated Magnetic Nanobeads in a** **Flow Enrichment Target Capture Halbach (FETCH) Magnetic Separation System for Circulating Tumor Cells Enrichment**

Peng Liu ^1,2‡^, Sitian He ^1,3‡^, Anouk Mentink^1^, Pieter Hart ^1^, Yongjun Wu ^3^, Leon WMM Terstappen ^1,4^, Pascal Jonkheijm ^2*^, Michiel Stevens ^1,5*^

‡ These authors contributed equally to this work.

1. Department of Medical Cell Biophysics, TechMed Center, Faculty of Science and Technology, University of Twente, PO Box 217, 7500 AE Enschede, The Netherlands.
2. Department of Molecules and Materials, Laboratory of Biointerface Chemistry and the TechMed Centre, University of Twente, Enschede, The Netherlands.
3. College of Public Health, Zhengzhou University, Zhengzhou, China.
4. Department of General, Visceral and Pediatric Surgery, Heinrich-Heine University, University Hospital Düsseldorf, 40225 Düsseldorf, Germany.
5. FETCH BV, Deventer, The Netherlands

*Corresponding authors: Pascal Jonkheijm email: p.jonkheijm@utwente.nl, Phone: +31534892987 & Michiel Stevens email: m.stevens@utwente.nl, Phone.: +31534894101


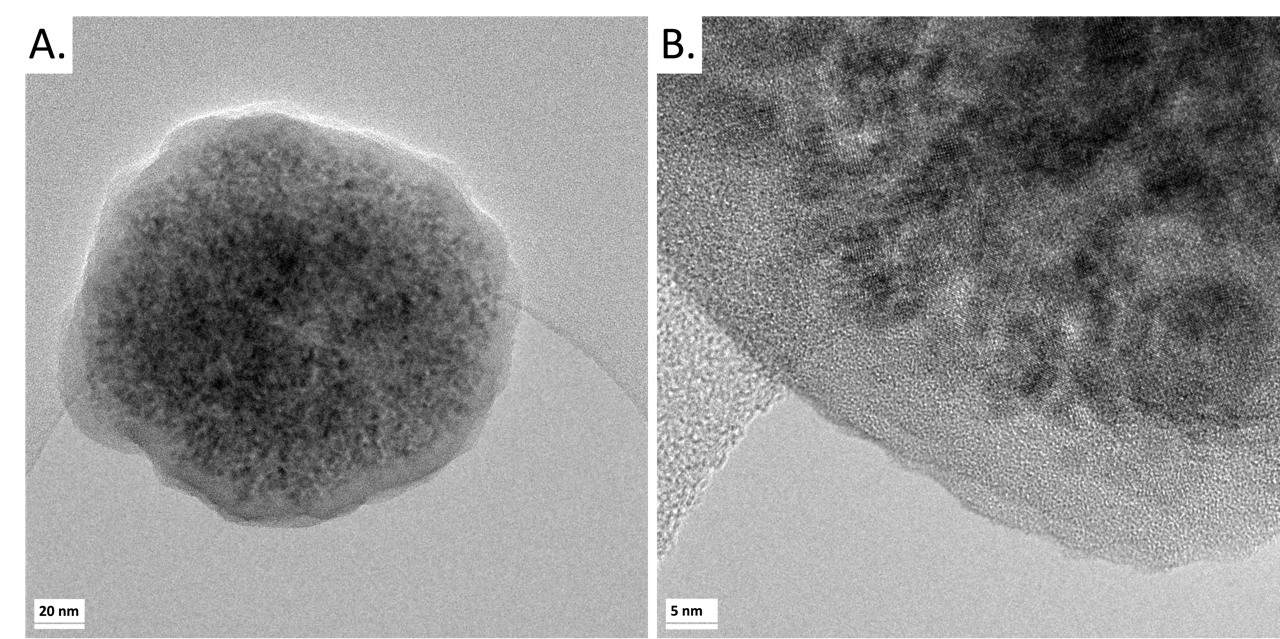


Figure S1. Transmission electron microscopy (TEM) images of NC@silica beads. (A) TEM image of an NC@silica bead, showing its overall morphology with a scale bar of 20 nm; (B) Enlarged TEM image highlighting the silica shell structure of an NC@silica bead, with a scale bar of 5 nm.


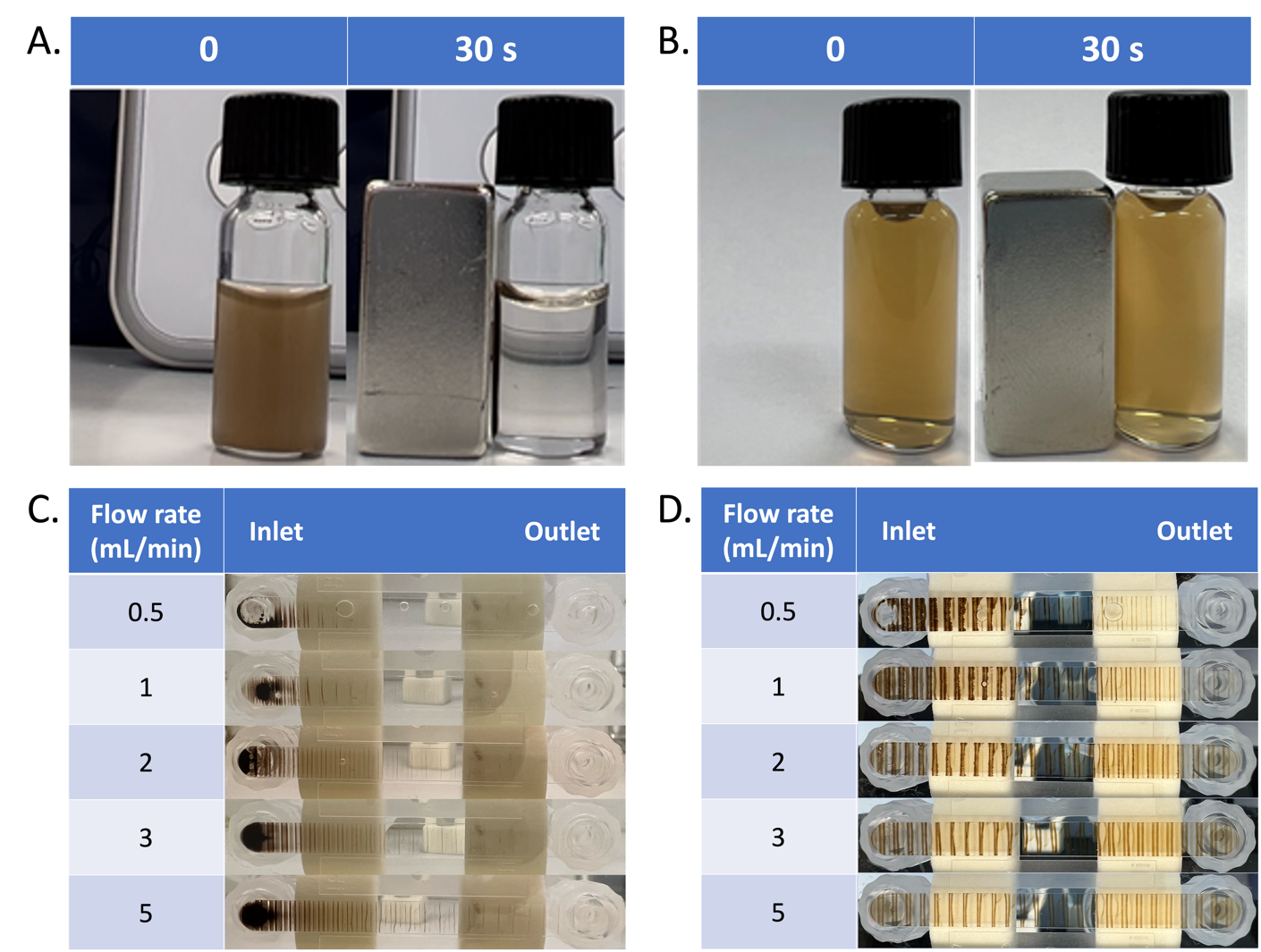


Figure S2. (A) Physical images depict NC@silica-SA beads being magnetically captured at various time intervals. (B) A physical image illustrates Mojosort beads being magnetically captured at different time points. (C) Physical images show NC@silica-SA beads captured by the FETCH system at different time intervals. (D) Physical images display Mojosort beads captured by the FETCH system at various times.


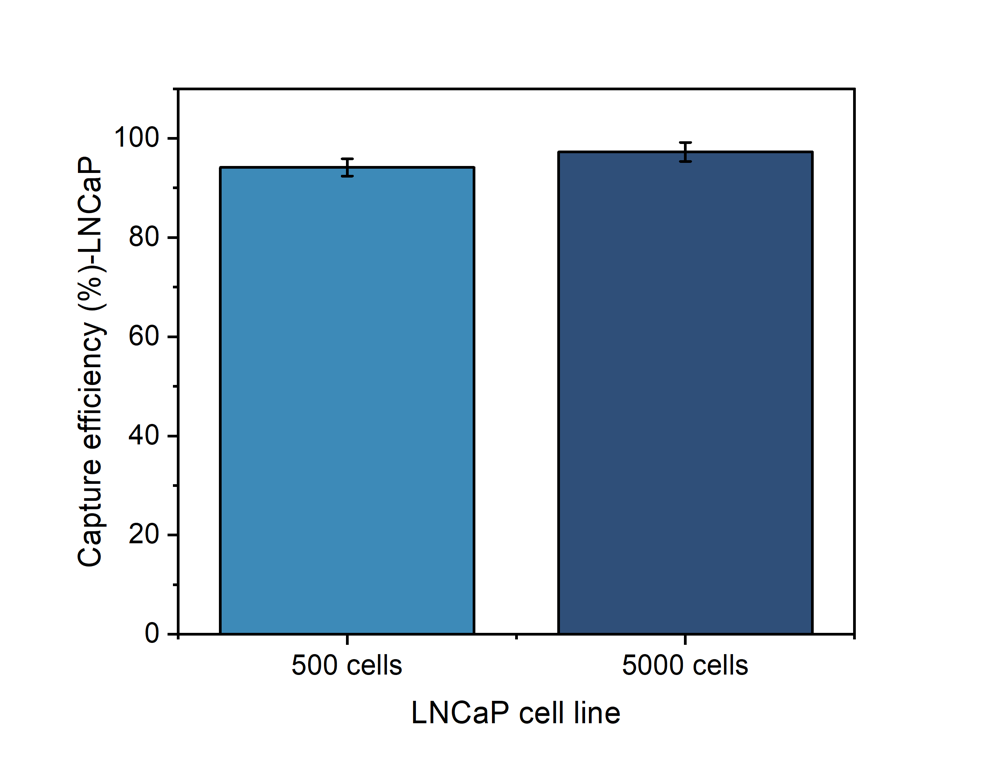


Figure S3. Capture efficiency comparison of spike in 500 and 5000 LNCaP cells. n=3. Columns indicate the mean, whiskers indicate the Mean ± SD.
